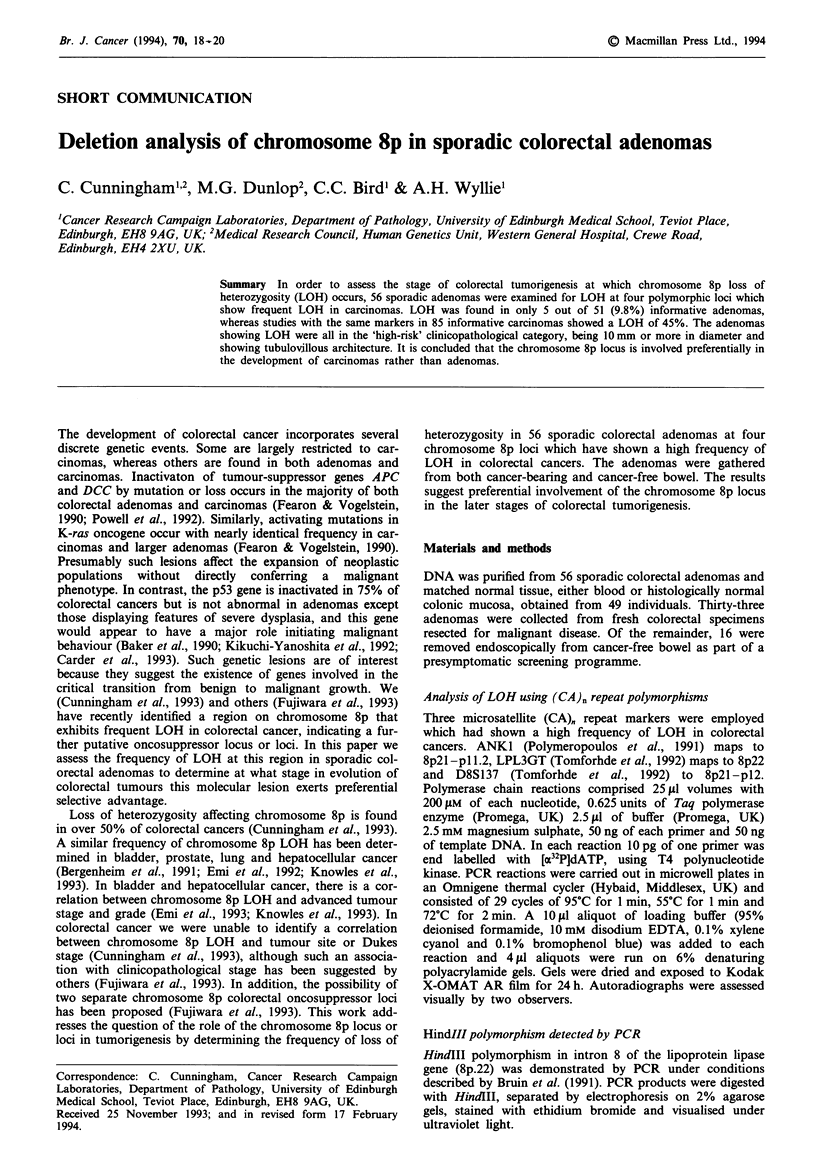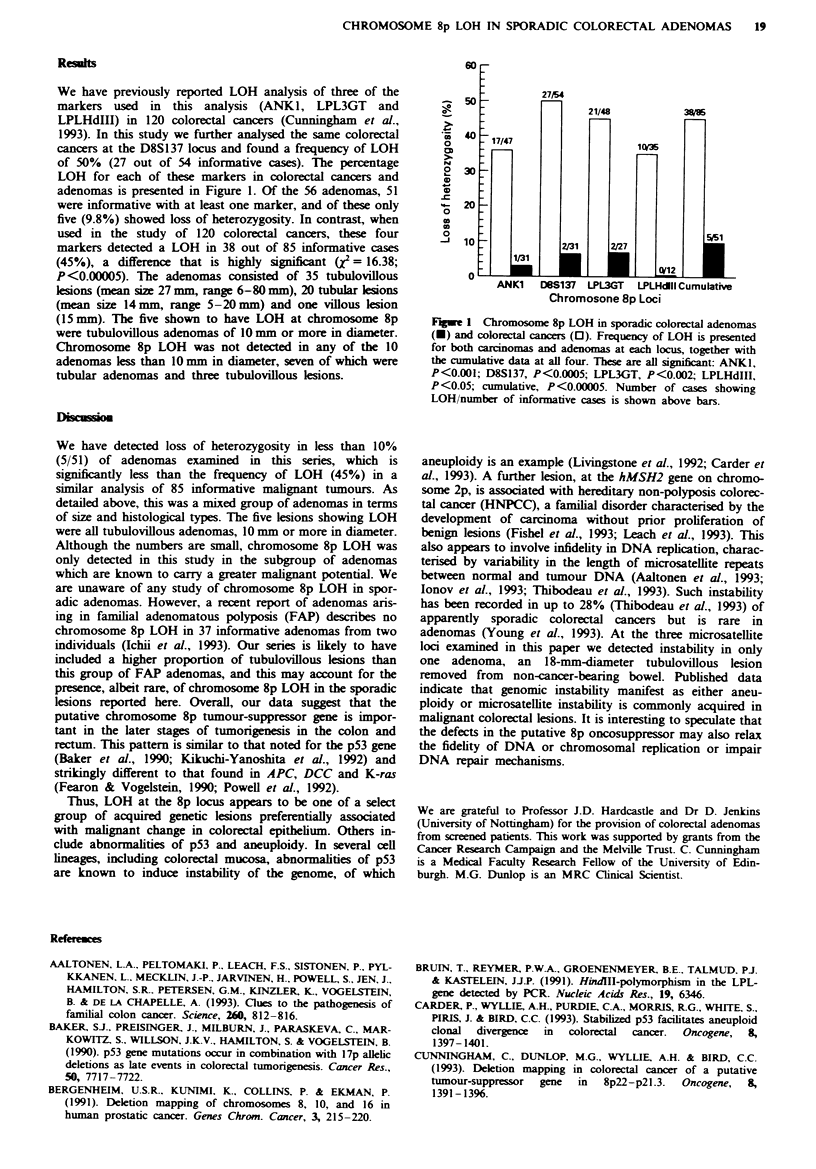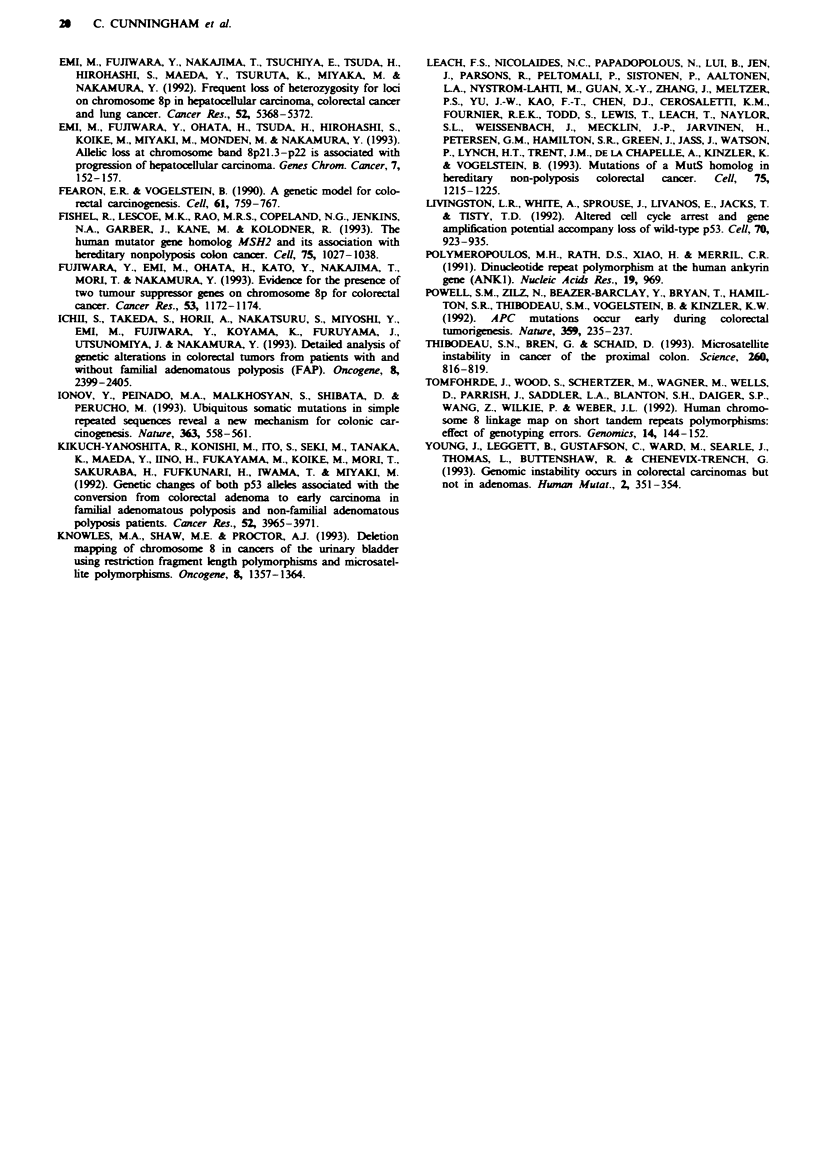# Deletion analysis of chromosome 8p in sporadic colorectal adenomas.

**DOI:** 10.1038/bjc.1994.243

**Published:** 1994-07

**Authors:** C. Cunningham, M. G. Dunlop, C. C. Bird, A. H. Wyllie

**Affiliations:** Department of Pathology, University of Edinburgh Medical School, UK.

## Abstract

In order to assess the stage of colorectal tumorigenesis at which chromosome 8p loss of heterozygosity (LOH) occurs, 56 sporadic adenomas were examined for LOH at four polymorphic loci which show frequent LOH in carcinomas. LOH was found in only 5 out of 51 (9.8%) informative adenomas, whereas studies with the same markers in 85 informative carcinomas showed a LOH of 45%. The adenomas showing LOH were all in the 'high-risk' clinicopathological category, being 10 mm or more in diameter and showing tubulovillous architecture. It is concluded that the chromosome 8p locus is involved preferentially in the development of carcinomas rather than adenomas.


Br. J. Cancer (1994), 70, 18–20 © Macmillan Press Ltd., 1994

SHORT COMMUNICATION

### Deletion analysis of chromosome 8p in sporadic colorectal adenomas

C. Cunningham[1,2], M.G. Dunlop[2], C.C. Bird[1] & A.H. Wyllie[1]

[1]*Cancer Research Campaign Laboratories, Department of Pathology, University of Edinburgh Medical School, Teviot Place, Edinburgh, EH8 9AG, UK;* [2]*Medical Research Council, Human Genetics Unit, Western General Hospital, Crewe Road, Edinburgh, EH4 2XU, UK.*

**Summary** In order to assess the stage of colorectal tumorigenesis at which chromosome 8p loss of heterozygosity (LOH) occurs, 56 sporadic adenomas were examined for LOH at four polymorphic loci which show frequent LOH in carcinomas. LOH was found in only 5 out of 51 (9.8%) informative adenomas, whereas studies with the same markers in 85 informative carcinomas showed a LOH of 45%. The adenomas showing LOH were all in the 'high-risk' clinicopathological category, being 10 mm or more in diameter and showing tubulovillous architecture. It is concluded that the chromosome 8p locus is involved preferentially in the development of carcinomas rather than adenomas.

The development of colorectal cancer incorporates several discrete genetic events. Some are largely restricted to carcinomas, whereas others are found in both adenomas and carcinomas. Inactivaton of tumour-suppressor genes *APC* and *DCC* by mutation or loss occurs in the majority of both colorectal adenomas and carcinomas (Fearon & Vogelstein, 1990; Powell *et al.*, 1992). Similarly, activating mutations in K-*ras* oncogene occur with nearly identical frequency in carcinomas and larger adenomas (Fearon & Vogelstein, 1990). Presumably such lesions affect the expansion of neoplastic populations without directly conferring a malignant phenotype. In contrast, the p53 gene is inactivated in 75% of colorectal cancers but is not abnormal in adenomas except those displaying features of severe dysplasia, and this gene would appear to have a major role initiating malignant behaviour (Baker *et al.*, 1990; Kikuchi-Yanoshita *et al.*, 1992; Carder *et al.*, 1993). Such genetic lesions are of interest because they suggest the existence of genes involved in the critical transition from benign to malignant growth. We (Cunningham *et al.*, 1993) and others (Fujiwara *et al.*, 1993) have recently identified a region on chromosome 8p that exhibits frequent LOH in colorectal cancer, indicating a further putative oncosuppressor locus or loci. In this paper we assess the frequency of LOH at this region in sporadic colorectal adenomas to determine at what stage in evolution of colorectal tumours this molecular lesion exerts preferential selective advantage.

Loss of heterozygosity affecting chromosome 8p is found in over 50% of colorectal cancers (Cunningham *et al.*, 1993). A similar frequency of chromosome 8p LOH has been determined in bladder, prostate, lung and hepatocellular cancer (Bergenheim *et al.*, 1991; Emi *et al.*, 1992; Knowles *et al.*, 1993). In bladder and hepatocellular cancer, there is a correlation between chromosome 8p LOH and advanced tumour stage and grade (Emi *et al.*, 1993; Knowles *et al.*, 1993). In colorectal cancer we were unable to identify a correlation between chromosome 8p LOH and tumour site or Dukes stage (Cunningham *et al.*, 1993), although such an association with clinicopathological stage has been suggested by others (Fujiwara *et al.*, 1993). In addition, the possibility of two separate chromosome 8p colorectal oncosuppressor loci has been proposed (Fujiwara *et al.*, 1993). This work addresses the question of the role of the chromosome 8p locus or loci in tumorigenesis by determining the frequency of loss of heterozygosity in 56 sporadic colorectal adenomas at four chromosome 8p loci which have shown a high frequency of LOH in colorectal cancers. The adenomas were gathered from both cancer-bearing and cancer-free bowel. The results suggest preferential involvement of the chromosome 8p locus in the later stages of colorectal tumorigenesis.

Correspondence: C. Cunningham, Cancer Research Campaign Laboratories, Department of Pathology, University of Edinburgh Medical School, Teviot Place, Edinburgh, EH8 9AG, UK.
Received 25 November 1993; and in revised form 17 February 1994.

### Materials and methods

DNA was purified from 56 sporadic colorectal adenomas and matched normal tissue, either blood or histologically normal colonic mucosa, obtained from 49 individuals. Thirty-three adenomas were collected from fresh colorectal specimens resected for malignant disease. Of the remainder, 16 were removed endoscopically from cancer-free bowel as part of a presymptomatic screening programme.

### *Analysis of LOH using* $(CA)_n$ *repeat polymorphisms*

Three microsatellite $(CA)_n$ repeat markers were employed which had shown a high frequency of LOH in colorectal cancers. ANK1 (Polymeropoulos *et al.*, 1991) maps to 8p21–p11.2, LPL3GT (Tomforhde *et al.*, 1992) maps to 8p22 and D8S137 (Tomforhde *et al.*, 1992) to 8p21–p12. Polymerase chain reactions comprised 25 µl volumes with 200 µM of each nucleotide, 0.625 units of *Taq* polymerase enzyme (Promega, UK) 2.5 µl of buffer (Promega, UK) 2.5 mM magnesium sulphate, 50 ng of each primer and 50 ng of template DNA. In each reaction 10 pg of one primer was end labelled with [$\alpha$-$^{32}$P]dATP, using T4 polynucleotide kinase. PCR reactions were carried out in microwell plates in an Omnigene thermal cycler (Hybaid, Middlesex, UK) and consisted of 29 cycles of 95°C for 1 min, 55°C for 1 min and 72°C for 2 min. A 10 µl aliquot of loading buffer (95% deionised formamide, 10 mM disodium EDTA, 0.1% xylene cyanol and 0.1% bromophenol blue) was added to each reaction and 4 µl aliquots were run on 6% denaturing polyacrylamide gels. Gels were dried and exposed to Kodak X-OMAT AR film for 24 h. Autoradiographs were assessed visually by two observers.

### Hind*III polymorphism detected by PCR*

*Hind*III polymorphism in intron 8 of the lipoprotein lipase gene (8p.22) was demonstrated by PCR under conditions described by Bruin *et al.* (1991). PCR products were digested with *Hind*III, separated by electrophoresis on 2% agarose gels, stained with ethidium bromide and visualised under ultraviolet light.

### Results

We have previously reported LOH analysis of three of the markers used in this analysis (ANK1, LPL3GT and LPLHdIII) in 120 colorectal cancers (Cunningham *et al.*, 1993). In this study we further analysed the same colorectal cancers at the D8S137 locus and found a frequency of LOH of 50% (27 out of 54 informative cases). The percentage LOH for each of these markers in colorectal cancers and adenomas is presented in Figure 1. Of the 56 adenomas, 51 were informative with at least one marker, and of these only five (9.8%) showed loss of heterozygosity. In contrast, when used in the study of 120 colorectal cancers, these four markers detected a LOH in 38 out of 85 informative cases (45%), a difference that is highly significant ($\chi^2 = 16.38$; $P < 0.00005$). The adenomas consisted of 35 tubulovillous lesions (mean size 27 mm, range 6–80 mm), 20 tubular lesions (mean size 14 mm, range 5–20 mm) and one villous lesion (15 mm). The five shown to have LOH at chromosome 8p were tubulovillous adenomas of 10 mm or more in diameter. Chromosome 8p LOH was not detected in any of the 10 adenomas less than 10 mm in diameter, seven of which were tubular adenomas and three tubulovillous lesions.

**Figure 1** Chromosome 8p LOH in sporadic colorectal adenomas (■) and colorectal cancers (□). Frequency of LOH is presented for both carcinomas and adenomas at each locus, together with the cumulative data at all four. These are all significant: ANK1, $P < 0.001$; D8S137, $P < 0.0005$; LPL3GT, $P < 0.002$; LPLHdIII, $P < 0.05$; cumulative, $P < 0.00005$. Number of cases showing LOH/number of informative cases is shown above bars.

### Discussion

We have detected loss of heterozygosity in less than 10% (5/51) of adenomas examined in this series, which is significantly less than the frequency of LOH (45%) in a similar analysis of 85 informative malignant tumours. As detailed above, this was a mixed group of adenomas in terms of size and histological types. The five lesions showing LOH were all tubulovillous adenomas, 10 mm or more in diameter. Although the numbers are small, chromosome 8p LOH was only detected in this study in the subgroup of adenomas which are known to carry a greater malignant potential. We are unaware of any study of chromosome 8p LOH in sporadic adenomas. However, a recent report of adenomas arising in familial adenomatous polyposis (FAP) describes no chromosome 8p LOH in 37 informative adenomas from two individuals (Ichii *et al.*, 1993). Our series is likely to have included a higher proportion of tubulovillous lesions than this group of FAP adenomas, and this may account for the presence, albeit rare, of chromosome 8p LOH in the sporadic lesions reported here. Overall, our data suggest that the putative chromosome 8p tumour-suppressor gene is important in the later stages of tumorigenesis in the colon and rectum. This pattern is similar to that noted for the p53 gene (Baker *et al.*, 1990; Kikuchi-Yanoshita *et al.*, 1992) and strikingly different to that found in *APC*, *DCC* and K-*ras* (Fearon & Vogelstein, 1990; Powell *et al.*, 1992).

Thus, LOH at the 8p locus appears to be one of a select group of acquired genetic lesions preferentially associated with malignant change in colorectal epithelium. Others include abnormalities of p53 and aneuploidy. In several cell lineages, including colorectal mucosa, abnormalities of p53 are known to induce instability of the genome, of which aneuploidy is an example (Livingstone *et al.*, 1992; Carder *et al.*, 1993). A further lesion, at the *hMSH2* gene on chromosome 2p, is associated with hereditary non-polyposis colorectal cancer (HNPCC), a familial disorder characterised by the development of carcinoma without prior proliferation of benign lesions (Fishel *et al.*, 1993; Leach *et al.*, 1993). This also appears to involve infidelity in DNA replication, characterised by variability in the length of microsatellite repeats between normal and tumour DNA (Aaltonen *et al.*, 1993; Ionov *et al.*, 1993; Thibodeau *et al.*, 1993). Such instability has been recorded in up to 28% (Thibodeau *et al.*, 1993) of apparently sporadic colorectal cancers but is rare in adenomas (Young *et al.*, 1993). At the three microsatellite loci examined in this paper we detected instability in only one adenoma, an 18-mm-diameter tubulovillous lesion removed from non-cancer-bearing bowel. Published data indicate that genomic instability manifest as either aneuploidy or microsatellite instability is commonly acquired in malignant colorectal lesions. It is interesting to speculate that the defects in the putative 8p oncosuppressor may also relax the fidelity of DNA or chromosomal replication or impair DNA repair mechanisms.

We are grateful to Professor J.D. Hardcastle and Dr D. Jenkins (University of Nottingham) for the provision of colorectal adenomas from screened patients. This work was supported by grants from the Cancer Research Campaign and the Melville Trust. C. Cunningham is a Medical Faculty Research Fellow of the University of Edinburgh. M.G. Dunlop is an MRC Clinical Scientist.

### References

AALTONEN, L.A., PELTOMAKI, P., LEACH, F.S., SISTONEN, P., PYLKKANEN, L., MECKLIN, J.-P., JARVINEN, H., POWELL, S., JEN, J., HAMILTON, S.R., PETERSEN, G.M., KINZLER, K., VOGELSTEIN, B. & DE LA CHAPELLE, A. (1993). Clues to the pathogenesis of familial colon cancer. *Science*, **260**, 812–816.

BAKER, S.J., PREISINGER, J., MILBURN, J., PARASKEVA, C., MARKOWITZ, S., WILLSON, J.K.V., HAMILTON, S. & VOGELSTEIN, B. (1990). p53 gene mutations occur in combination with 17p allelic deletions as late events in colorectal tumorigenesis. *Cancer Res.*, **50**, 7717–7722.

BERGENHEIM, U.S.R., KUNIMI, K., COLLINS, P. & EKMAN, P. (1991). Deletion mapping of chromosomes 8, 10, and 16 in human prostatic cancer. *Genes Chrom. Cancer*, **3**, 215–220.

BRUIN, T., REYMER, P.W.A., GROENENMEYER, B.E., TALMUD, P.J. & KASTELEIN, J.J.P. (1991). *Hind*III-polymorphism in the LPL-gene detected by PCR. *Nucleic Acids Res.*, **19**, 6346.

CARDER, P., WYLLIE, A.H., PURDIE, C.A., MORRIS, R.G., WHITE, S., PIRIS, J. & BIRD, C.C. (1993). Stabilized p53 facilitates aneuploid clonal divergence in colorectal cancer. *Oncogene*, **8**, 1397–1401.

CUNNINGHAM, C., DUNLOP, M.G., WYLLIE, A.H. & BIRD, C.C. (1993). Deletion mapping in colorectal cancer of a putative tumour-suppressor gene in 8p22–p21.3. *Oncogene*, **8**, 1391–1396.

EMI, M., FUJIWARA, Y., NAKAJIMA, T., TSUCHIYA, E., TSUDA, H., HIROHASHI, S., MAEDA, Y., TSURUTA, K., MIYAKA, M. & NAKAMURA, Y. (1992). Frequent loss of heterozygosity for loci on chromosome 8p in hepatocellular carcinoma, colorectal cancer and lung cancer. *Cancer Res.*, **52**, 5368–5372.

EMI, M., FUJIWARA, Y., OHATA, H., TSUDA, H., HIROHASHI, S., KOIKE, M., MIYAKI, M., MONDEN, M. & NAKAMURA, Y. (1993). Allelic loss at chromosome band 8p21.3–p22 is associated with progression of hepatocellular carcinoma. *Genes Chrom. Cancer*, **7**, 152–157.

FEARON, E.R. & VOGELSTEIN, B. (1990). A genetic model for colorectal carcinogenesis. *Cell*, **61**, 759–767.

FISHEL, R., LESCOE, M.K., RAO, M.R.S., COPELAND, N.G., JENKINS, N.A., GARBER, J., KANE, M. & KOLODNER, R. (1993). The human mutator gene homolog *MSH2* and its association with hereditary nonpolyposis colon cancer. *Cell*, **75**, 1027–1038.

FUJIWARA, Y., EMI, M., OHATA, H., KATO, Y., NAKAJIMA, T., MORI, T. & NAKAMURA, Y. (1993). Evidence for the presence of two tumour suppressor genes on chromosome 8p for colorectal cancer. *Cancer Res.*, **53**, 1172–1174.

ICHII, S., TAKEDA, S., HORII, A., NAKATSURU, S., MIYOSHI, Y., EMI, M., FUJIWARA, Y., KOYAMA, K., FURUYAMA, J., UTSUNOMIYA, J. & NAKAMURA, Y. (1993). Detailed analysis of genetic alterations in colorectal tumors from patients with and without familial adenomatous polyposis (FAP). *Oncogene*, **8**, 2399–2405.

IONOV, Y., PEINADO, M.A., MALKHOSYAN, S., SHIBATA, D. & PERUCHO, M. (1993). Ubiquitous somatic mutations in simple repeated sequences reveal a new mechanism for colonic carcinogenesis. *Nature*, **363**, 558–561.

KIKUCH-YANOSHITA, R., KONISHI, M., ITO, S., SEKI, M., TANAKA, K., MAEDA, Y., IINO, H., FUKAYAMA, M., KOIKE, M., MORI, T., SAKURABA, H., FUFKUNARI, H., IWAMA, T. & MIYAKI, M. (1992). Genetic changes of both p53 alleles associated with the conversion from colorectal adenoma to early carcinoma in familial adenomatous polyposis and non-familial adenomatous polyposis patients. *Cancer Res.*, **52**, 3965–3971.

KNOWLES, M.A., SHAW, M.E. & PROCTOR, A.J. (1993). Deletion mapping of chromosome 8 in cancers of the urinary bladder using restriction fragment length polymorphisms and microsatellite polymorphisms. *Oncogene*, **8**, 1357–1364.

LEACH, F.S., NICOLAIDES, N.C., PAPADOPOLOUS, N., LUI, B., JEN, J., PARSONS, R., PELTOMALI, P., SISTONEN, P., AALTONEN, L.A., NYSTROM-LAHTI, M., GUAN, X.-Y., ZHANG, J., MELTZER, P.S., YU, J.-W., KAO, F.-T., CHEN, D.J., CEROSALETTI, K.M., FOURNIER, R.E.K., TODD, S., LEWIS, T., LEACH, T., NAYLOR, S.L., WEISSENBACH, J., MECKLIN, J.-P., JARVINEN, H., PETERSEN, G.M., HAMILTON, S.R., GREEN, J., JASS, J., WATSON, P., LYNCH, H.T., TRENT, J.M., DE LA CHAPELLE, A., KINZLER, K. & VOGELSTEIN, B. (1993). Mutations of a MutS homolog in hereditary non-polyposis colorectal cancer. *Cell*, **75**, 1215–1225.

LIVINGSTON, L.R., WHITE, A., SPROUSE, J., LIVANOS, E., JACKS, T. & TISTY, T.D. (1992). Altered cell cycle arrest and gene amplification potential accompany loss of wild-type p53. *Cell*, **70**, 923–935.

POLYMEROPOULOS, M.H., RATH, D.S., XIAO, H. & MERRIL, C.R. (1991). Dinucleotide repeat polymorphism at the human ankyrin gene (ANK1). *Nucleic Acids Res.*, **19**, 969.

POWELL, S.M., ZILZ, N., BEAZER-BARCLAY, Y., BRYAN, T., HAMILTON, S.R., THIBODEAU, S.M., VOGELSTEIN, B. & KINZLER, K.W. (1992). *APC* mutations occur early during colorectal tumorigenesis. *Nature*, **359**, 235–237.

THIBODEAU, S.N., BREN, G. & SCHAID, D. (1993). Microsatellite instability in cancer of the proximal colon. *Science*, **260**, 816–819.

TOMFOHRDE, J., WOOD, S., SCHERTZER, M., WAGNER, M., WELLS, D., PARRISH, J., SADDLER, L.A., BLANTON, S.H., DAIGER, S.P., WANG, Z., WILKIE, P. & WEBER, J.L. (1992). Human chromosome 8 linkage map on short tandem repeats polymorphisms: effect of genotyping errors. *Genomics*, **14**, 144–152.

YOUNG, J., LEGGETT, B., GUSTAFSON, C., WARD, M., SEARLE, J., THOMAS, L., BUTTENSHAW, R. & CHENEVIX-TRENCH, G. (1993). Genomic instability occurs in colorectal carcinomas but not in adenomas. *Human Mutat.*, **2**, 351–354.